# Delayed Diagnosis of Congenital Hypothyroidism in a Child with Trisomy 21 and Biotinidase Deficiency and Successful Use of Levothyroxine Sodium Oral Solution

**DOI:** 10.1155/2020/8883969

**Published:** 2020-12-23

**Authors:** Matthew M. Feldt

**Affiliations:** Pediatric Endocrinologist, Children's Mercy Hospital, Kansas City, MO, USA

## Abstract

Endocrine disorders are more common and appear earlier in people with trisomy 21 (T21) than in the general population, with thyroid dysfunction being the most common, including both congenital and acquired hypothyroidism. The treatment for biotinidase deficiency, a condition that occurs in approximately 1 : 110,000 people, is with biotin (vitamin B7) supplementation. However, biotin can interfere with endocrine laboratory assays and cause falsely low thyroid-stimulating hormone (TSH) and elevated free thyroxine (FT4) levels. This can interfere with the timely diagnosis and subsequent treatment of congenital hypothyroidism (CH). This case report describes an infant with partial biotinidase deficiency that was confirmed on day 10 of life. Routine screening erroneously reported “normal” TSH that caused delayed diagnosis of CH due to interference with the TSH assay from concurrent biotin use. Once the biotin treatment was withheld for 4 days and the thyroid function tests repeated, an elevated TSH became apparent. Treatment with tablet levothyroxine (L-T4) was started and subsequently changed to L-T4 oral solution (Tirosint®-SOL) to overcome treatment administration difficulties encountered with the tablet form. This resulted in improved TSH control due to more accurate and consistent dosing compared with the tablet formulation. This is the first report of the use of L-T4 oral solution in an infant with T21 and biotinidase deficiency.

## 1. Introduction

Trisomy 21 (T21), or Down syndrome, affects both the physical and intellectual development of the individual [1, 2]. Endocrine disorders are more common and appear earlier in people with T21 than in the general population [3]. Of the many endocrine disorders associated with T21, thyroid dysfunction is the most common and includes both congenital and acquired hypothyroidism [3–6]. Biotinidase deficiency is a relatively rare condition occurring in approximately 1 : 110,000 [7]. Biotin (vitamin B7) supplementation is the treatment in biotinidase deficiency but can interfere with many laboratory assays [8], causing falsely low levels of thyroid-stimulating hormone (TSH) and elevated free thyroxine (FT4) levels [9]. This can interfere with the timely diagnosis of congenital hypothyroidism (CH) [10]. Levothyroxine (L-T4), in tablet form, is considered the standard of care for treatment of hypothyroidism [11].

The therapeutic administration of tablet L-T4 in patients with T21 may prove challenging due to the often-associated occurrence of global developmental delay [2] and gastrointestinal problems, including functional and anatomical anomalies [1]. There is a paucity of literature about concomitant CH and biotinidase deficiency, and no prior literature describing the use of levothyroxine sodium oral solution (L-T4 oral solution), Tirosint®-SOL, which has recently become available in the United States for the treatment of hypothyroidism [12]. Prior European studies on the use of L-T4 oral solution in CH have shown initial improvements in TSH and/or maintenance of euthyroidism over time versus tablets [13–15]. These observations may be linked to higher absorption and bioequivalence for liquid L-T4 solution compared with tablets [13–15].

This case report describes a patient with “normal” TSH on routine screening that caused delayed diagnosis of CH due to interference with the TSH assay from concurrent biotin use. Once biotin treatment was withheld for 4 days and thyroid function tests repeated, an elevated TSH became apparent and CH was diagnosed. Treatment with tablet L-T4 was started and subsequently changed to L-T4 oral solution to overcome administration difficulties. This resulted in easier administration, accurate dosing, improved compliance, and biochemical euthyroidism.

## 2. Case Presentation

**Figure 1:**
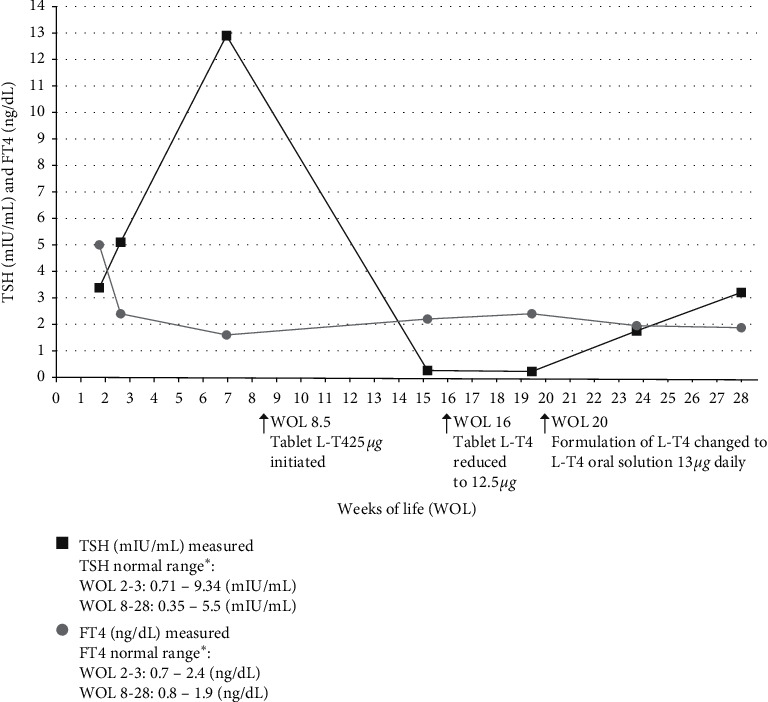
Thyroid-stimulating hormone (TSH) and free thyroxine (FT4) timeline. ^*∗*^Normal ranges shown are those used by the author's institution. L-T4, levothyroxine sodium oral solution; WOL, weeks of life.

The patient is a white, non-Hispanic, full-term, infant female with T21. Pertinent family history includes both parents being confirmed carriers for biotinidase deficiency. TSH-based newborn screening was performed and not flagged as abnormal for hypothyroidism. The patient was confirmed with partial biotinidase deficiency (2.0 U/L; reference range: 3.5–13.8 U/L) on day of life 10 and started on biotin 1000 *µ*g daily. At 2 weeks of life (WOL), thyroid function was tested while receiving biotin: TSH was 3.39 mIU/mL and FT4 was elevated at 5 ng/dL. Autoimmune evaluations for hyperthyroidism in the mother and child were negative. At 3 WOL, biotin was withheld for 3 days and repeat thyroid function tests showed a TSH of 5.1 mIU/mL and that FT4 had normalized to 2.4 mIU/mL. At 8 WOL, after biotin was withheld for 4 days, TSH was now elevated and FT4 remained in the reference range. Congenital hypothyroidism was diagnosed at that time and the child was started on the tablet form of L-T4 25 *µ*g. The patient's parents reported difficulty in administering the dose via a crushed tablet in a bottle of formula. At 16 WOL, biotin was withheld for 4 days and repeat thyroid function tests showed that TSH was suppressed and FT4 was slightly elevated. The dose of L-T4 was reduced to 12.5 *µ*g (half tablet) and at 20 WOL, after biotin was withheld for 4 days, repeat thyroid testing again showed suppressed TSH and elevated FT4. The parents still reported difficulty with cutting and crushing the L-T4 tablet, resulting in inconsistent dosing. A referral to pediatric endocrine care was then initiated and the L-T4 tablet formulation was changed to oral solution 13 *µ*g daily. The parents reported that administration was much easier with the oral formulation. At 24 WOL, biotin was again withheld for 4 days and repeat thyroid function tests showed normalization in both TSH (1.75 mIU/mL) and FT4 (1.96 ng/dL). At 28 WOL, after biotin was withheld for 4 days, both TSH and FT4 were still normal and the family reported being very happy with the L-T4 oral solution. A timeline of treatments administered along with outcomes is shown in Table 1 and Figure 1.

## 3. Discussion

The prognosis for children with CH has improved significantly due to the implementation of neonatal screening methods and a more focused therapeutic approach [16]. Studies on cognitive function in patients with CH treated soon after birth have shown that normal development can be achieved in most patients, although cognitive development depends on the severity of the disorder and the age at which the hormone replacement therapy with L-T4 is started [16, 17]. Most neonatal screening methods in the United States use a combination of TSH, FT4, or both [18].

Biotinidase deficiency, the major cause of late-onset biotin-responsive multiple carboxylase deficiency, is an autosomal recessive neurocutaneous disorder [7]. The symptoms of biotinidase deficiency can be successfully treated or prevented by administering pharmacological doses of biotin [7], a water-soluble vitamin used widely as a dietary supplement. In children with biotinidase deficiency, doses are considerably higher (5-10 mg per kilogram of body weight per day) [7] than the normal dietary reference intake for children (5–25 *μ*g per kilogram per day) [19]. Biotin has been shown to interfere with many endocrine laboratory assays (including TSH and FT4) [8, 9], which can lead to erroneous or delayed diagnosis of CH. Prior literature has described cases of children receiving an inaccurate diagnosis of hyperthyroidism due to interference with the thyroid function assay from high doses of biotin being given as a therapy for metabolic disease [9, 10]. Our patient with biotinidase deficiency had erroneous thyroid function testing that was suggestive of hyperthyroidism. However, once biotin supplementation was withheld, a true diagnosis of CH was possible.

Thyroid hormones are pivotal for normal physical and neuromotor development during the first years of life. Compliance with L-T4 treatment is one of the main factors that influence the outcome of patients with CH [20]. The therapeutic use of biotin in this infant resulted in false TSH assay measurements so that they appeared in the normal range and delayed the diagnosis of CH. Once the correct diagnosis was made and treatment initiated, the TSH and FT4 levels still remained outside the reference range because of the suboptimal administration of tablet-formulated L-T4, which should have been given in crushed form via a syringe, but instead was being mixed with infant formula. However, changing to L-T4 oral solution improved the ease and consistency of administration and compliance with therapy, and TSH levels subsequently improved.

T21 may cause additional challenges in infants and young children receiving L-T4 treatment due to associated gastrointestinal problems, feeding difficulties, and developmental delays [1]. In addition to the obvious advantage of administering an oral solution instead of a crushed tablet to an infant, L-T4 oral solution has many advantages for this patient population: it has a sweet taste; contains only levothyroxine, glycerol, and water [12]; is free from additional excipients; and does not require a gastric phase of dissolution (before absorption), meaning that it is more readily absorbed than tablets [21]. Compared with the tablet form of LT-4, faster achievement of target TSH values was demonstrated for the oral form and it also allows for easier individualization of the dose [22]. As accurate dosing of L-T4, both at initiation and in the long term, has been recognized as a major challenge in the treatment of CH [22], this case demonstrates the utility of L-T4 oral solution in enabling ease of administration, accurate dosing, and subsequent TSH control in a patient with CH and T21.

In conclusion, this case demonstrates the effectiveness of using L-T4 oral solution to treat a patient with CH and T21. The use of L-T4 oral solution (Tirosint®-SOL) may improve compliance and consistency of thyroid control in pediatric patients, especially in patients with T21 with/or without CH who have special needs. Also, clinicians should be aware that infants receiving biotin supplementation for biotinidase deficiency will need this therapy to be withheld before screening for CH. Failure to do so can result in erroneous measurement of thyroid levels and delayed diagnosis and treatment of CH.

A limitation of this case report is that we report on a single subject who was followed for a short time. However, this case provides point-of-care evidence that could help to inform practice guidelines and provide practical medical education to other healthcare professionals.

## Figures and Tables

**Table 1:** Thyroid function tests after diagnosis of biotinidase deficiency.

Weeks of life	Days off biotin	TSH (mIU/mL)		FT4 (ng/dL)	
		Measured	Normal range^*∗*^	Measured	Normal range^*∗*^
2	0	3.39	0.71–9.34	5.0	0.7–2.4
3	3	5.1	0.71–9.34	2.4	0.7–2.4
8	4	12.9	0.35–5.5	1.6	0.8–1.9
8.5	Patient started on tablet L-T4 25 *µ*g				
16	3	0.08	0.35–5.5	2.2	0.8–1.9
	Dose of tablet L-T4 reduced to 12.5 *µ*g				
20	3	0.04	0.35–5.5	2.4	0.8–1.9
	Formulation of L-T4 changed to L-T4 oral solution 13 *µ*g daily				
24	4	1.75	0.35–5.5	1.96	0.8–1.9
28	4	3.2	0.35–5.5		0.8–1.9

^*∗*^Normal ranges shown are those used by the author's institution. FT4, free thyroxine; L-T4, levothyroxine; TSH, thyroid-stimulating hormone.

## Data Availability

Additional information pertaining to this case may be requested by contacting the corresponding author.
